# Early Antecedents of School Burnout in Upper Secondary Education: A Five-year Longitudinal Study

**DOI:** 10.1007/s10964-020-01331-w

**Published:** 2020-10-30

**Authors:** Milja Parviainen, Kaisa Aunola, Minna Torppa, Marja-Kristiina Lerkkanen, Anna-Maija Poikkeus, Kati Vasalampi

**Affiliations:** 1 Department of Psychology, Faculty of Education and Psychology, University of Jyväskylä, P.O. Box 35, 40014 Jyväskylä, Finland; 2 Department of Teacher Education, Faculty of Education and Psychology, University of Jyväskylä, P.O. Box 35, 40014 Jyväskylä, Finland

**Keywords:** School burnout, Academic skills, Psychological well-being, Upper secondary education, Developmental trajectories

## Abstract

School burnout symptoms are prevalent among upper secondary education students, but thus far, very little is known about the background of these symptoms. The present study examined the extent to which school burnout symptoms (i.e., exhaustion and cynicism) among upper secondary education students have their roots in primary and lower secondary school and whether early antecedents of school burnout symptoms could be identified. The sample consisted of 1544 Finnish students followed up four times (Time1–Time 4) from the end of primary school (T1; mean age 12.74 and range 11.71–14.20) to the first year of upper secondary education (T4; mean age 16.66 and range 15.55–18.39). The results of latent growth curve modeling showed that school burnout symptoms in upper secondary education were predicted by the level of school burnout symptoms at the end of primary school and by an increase in these symptoms across the transition from primary school through lower secondary school. In addition, psychological well-being, academic skills, and gender were found to contribute to the prediction of school burnout symptoms. Overall, the present study suggest that potential warning signs of school burnout should not be ignored and attention should be directed to earlier education phases.

## Introduction

Upper secondary education can constitute a particularly stressful period as it contains new challenges related to autonomous study and increased pressure to plan future educational and career paths. It is customary to experience schoolwork-related stress occasionally, but prolonged strain can lead to school burnout syndrome (Salmela-Aro et al. 2009a). School burnout among upper secondary education students has become a global concern (Walburg 2014). Finnish statistics (Finnish Institute for Health and Welfare 2019) indicate that almost one third of upper secondary education students in an academic track report experiencing relatively high levels of school-related exhaustion. Given the way school burnout is linked, for instance, to students’ poorer academic achievement (Madigan and Curran 2020), lower well-being (Salmela-Aro et al. 2009b), and dropping out of school (Bask and Salmela-Aro 2013), there is an evident need to prevent students from burning out. However, very little is known about the nature of the developmental trajectories and early antecedents that represent vulnerability to experiencing school burnout symptoms in upper secondary education. Therefore, the aim of this five-year longitudinal study was to examine the extent to which school burnout symptoms in upper secondary education have their roots in primary and lower secondary school and whether academic skills, psychological well-being, and gender are antecedents of school burnout symptoms.

### School Burnout Trajectories

Researchers first introduced burnout as a psychological syndrome resulting from chronic, unmanaged work-related stress (Maslach et al. 2001; World Health Organization 2018). Subsequently, researchers applied the construct of burnout to the school context drawing parallels between work stress and overtaxation due to studying and school work (Walburg 2014). Although several, partly overlapping concepts (e.g., school disengagement and school apathy) have been used in literature to describe maladjustment at school, the concept of school burnout offers a potential new insight into students’ school-related ill-being as it refers to persistent, multifaceted syndrome that can be evident as multiple different symptoms. Similarly to job burnout, school burnout is defined in terms of three dimensions: exhaustion, cynicism, and inadequacy (Schaufeli et al. 2002). According to Salmela-Aro et al. (2009a) exhaustion refers to school-related strain and chronic fatigue, cynicism describes loss of interest in schoolwork and an indifferent attitude to studying, and inadequacy refers to a reduced sense of accomplishment and students’ perception of being inadequate at school. The dimensions are distinct but closely linked (Salmela-Aro et al. 2009a), and thus these symptoms can be examined either as separate constructs or together as a single construct (i.e., overall school burnout). The former approach is supported by the studies that have shown that school burnout symptoms do not always go hand-in-hand: symptoms can be higher in one dimension than in another (Salmela-Aro et al. 2016; Tuominen-Soini and Salmela-Aro 2014). In the present study, two of the school burnout dimensions, that is, exhaustion and cynicism were investigated. Exhaustion is the stress component of burnout, whereas a cynical attitude is considered individuals’ way of distancing themselves from the overtaxing situation (Maslach et al. 2001).

Students’ psychological well-being fluctuates with the passing of school years especially during the school transitions (Salmela-Aro and Upadyaya 2014). In Finland, as in many countries, students experience two major educational transitions during adolescence: the first one (around 13 years of age) is from primary school to lower secondary school, and the second is from lower secondary school to upper secondary education (around 16 years of age) typically either to general upper secondary school (i.e., the theory-oriented academic track) or to vocational institutions (i.e., the practice-oriented vocational track). Researchers have reported that as students move further on their educational path school burnout symptoms seem to increase. Engels et al. (2019), for example, found that Finnish students showed somewhat higher levels of exhaustion in lower secondary school than at the end of primary school. Moreover, school burnout symptoms seem to keep increasing over the course of lower secondary school grades (Lee and Lee 2018; Salmela-Aro et al. 2018). During the transition to upper secondary education, school burnout symptoms have been shown to increase among students who choose the academic track over the vocational track (Salmela-Aro et al. 2008a; Salmela-Aro and Tynkkynen 2012), which is presumably due to the more demanding nature of academic track studies. However, although studies have detected some mean-level changes in school burnout symptoms by school year, the level of symptoms seems to be low and stable instead of fluctuating among most students during and after the transition from lower secondary school to upper secondary education (Salmela-Aro and Upadyaya 2014).

Although previous longitudinal studies shed some light on the development of school burnout symptoms, the studies typically covered only a short period. Therefore, analysis of longer periods is needed to unravel whether students’ burnout symptoms identified in upper secondary education develop for some during the course of the primary and lower secondary school years. Although scholars consider school burnout prevalent especially among older students (Lee et al. 2013), they have recognized it as a problem arising during the primary school years (Yang and Chen 2016). For example, evidence for school well-being among Finnish primary school students showed that nearly 40% of fourth and fifth graders often feel tired at school (Finnish Institute for Health and Welfare 2019), and nearly half of sixth graders experience some degree of cynicism (Salmela-Aro et al. 2016). Thus, it is possible that students reporting school burnout in upper secondary education may show signs of school burnout symptoms in primary school. However, thus far the extent to which school burnout symptoms identified in the earlier education phases predict subsequent symptoms in upper secondary education students is unclear.

### Antecedents of School Burnout

As longitudinal studies examining school burnout are scarce, it is not clear which factors are early indicators of the school burnout risk and predict school burnout symptoms in upper secondary education students. Therefore, understanding of individual-level factors that make students likely to experience school burnout symptoms is needed. Based on previous studies linking school burnout with various individual-level factors, it was assumed that basic academic skills (i.e., reading skills and arithmetic skills) (Korhonen et al. 2014; Widlund et al. 2018), psychological well-being and its problems (i.e., self-esteem and internalizing symptoms) (Kiuru et al. 2008; Parhiala et al. 2018), and gender (Salmela-Aro et al. 2008a) may be among the critical predictors of upper secondary education students’ school burnout symptoms.

Basic academic skills form the foundation for successful schoolwork, but thus far, relatively little is known about how reading and mathematical skills are related to students’ school burnout symptoms. Researchers have linked lower academic achievement to higher school burnout symptoms (Madigan and Curran 2020). However, most studies used grade point average or school grades as measures of skills. This is problematic, as these measures not only capture the skill level of an individual but may also tap the motivational aspects of performance, such as long-term persistence, task value, and interest in the school subject. In a rare study that used cognitive tests, May et al. (2015) showed that higher levels of school burnout symptoms are linked to poorer cognitive performance (i.e., problem solving and attentional/inhibition processes). Moreover, in a study using a person-centered approach, Korhonen et al. (2014) identified a group of students who had problems on reading, spelling, and mathematics tests who also reported symptoms of school burnout. Similarly, Widlund et al. (2018) identified a group of students with relatively low mathematics skills who showed relatively high levels of exhaustion, cynicism, and inadequacy. However, these studies also reported identifying a group of students with average skills and high levels of school burnout symptoms (Korhonen et al. 2014) and a group of students with poor skills combined with low levels of school burnout symptoms (Widlund et al. 2018) which suggest inconsistencies calling for further research on the links between school burnout and academic skills.

In addition to basic academic skills, psychological well-being can be considered to form a precondition for well-rounded school functioning. Problems in psychological well-being are known to accumulate: students with various psychological symptoms are likely to also experience burnout symptoms specific to the school context (Parviainen et al. 2020; Virtanen et al. 2019). Scholars have consistently found that internalizing symptoms, such as depressive symptoms, are linked to higher levels of school burnout symptoms (Fiorilli et al. 2017; Salmela-Aro et al. 2009b). Additionally, researchers have linked lower self-esteem, defined typically as the attitude toward oneself and a global evaluation of one’s self-worth (Baumeister et al. 2003; Rosenberg 1965), to higher levels of school burnout symptoms (Kiuru et al. 2008; Luo et al. 2016). Although there is evidence of the association between school burnout and internalizing symptoms and self-esteem, the extent to which the presence of these factors in early adolescence predicts trajectories of school burnout and symptoms in upper secondary education students is not yet known.

Additionally, researchers have shown that students’ gender plays a role in school burnout symptoms. Girls have been found to report higher levels of exhaustion than boys (e.g., Herrmann et al. 2019; Salmela-Aro et al. 2008a), which could stem from the high school-related pressure and demands that girls experience (Wiklund et al. 2012). Previous findings on gender differences in cynicism have, in contrast, been inconsistent: some studies showed that cynicism is higher among girls (Salmela-Aro et al. 2008a), but some studies did not detect gender differences (Cadime et al. 2016; Herrmann et al. 2019). Inconsistent findings in previous studies for the role of gender could be due to varying age groups and different educational contexts.

## Current Study

Due to the scarcity of longitudinal research on school burnout, very little is known about the developmental trajectories and antecedents that underlie upper secondary education students’ school burnout and how to recognize signs of school-burnout risk before students’ symptoms become exacerbated and prolonged. Although there are studies showing that academic skills (Korhonen et al. 2014), psychological well-being (Parhiala et al. 2018) and gender (Salmela-Aro et al. 2008a) are linked to school burnout symptoms, the studies focusing on these antecedents have mainly been cross-sectional. Consequently, little is known about the predictive role of these antecedents in subsequent school burnout symptoms. This five-year longitudinal study expands upon previous studies by identifying longitudinal trajectories and early antecedents of school burnout symptoms. The first aim of the study was to investigate the extent to which school burnout symptoms of exhaustion and cynicism as reported by students at the end of primary school (Grade 6) and the developmental trajectories from the end of primary school to the end of lower secondary school (Grades 6, 7, and 9) predict school burnout symptoms in the first year of upper secondary education. First, it was hypothesized (H1) that higher school burnout symptoms at the first measurement point, Grade 6, predict higher levels of symptoms in the first year of upper secondary education. Second, it was hypothesized (H2) that the more symptoms increase during the study period, the higher the levels of school burnout symptoms students show at the fourth measurement point in upper secondary education.

The second aim was to examine the extent to which different antecedent variables (i.e., psychological well-being in terms of self-esteem and internalizing symptoms and academic skills assessed with tests of reading and arithmetic skills) measured in Grade 6 as well as gender predict school burnout symptoms in the first year of upper secondary education either directly or indirectly via the level of school burnout symptoms in Grade 6 and via the developmental trajectories of symptoms (Grades 6–9). In line with previous documented links between school burnout symptoms and academic skills (Korhonen et al. 2014) and psychological well-being (Virtanen et al. 2019), it was hypothesized (H3) that lower levels of reading and arithmetic skills, and self-esteem and higher levels of internalizing symptoms predict higher levels of exhaustion and cynicism in upper secondary education students either directly or indirectly via the initial level and the developmental trajectories. It was also hypothesized (H4) that girls report higher levels of exhaustion in upper secondary education, whereas no gender differences were expected in cynicism (Herrmann et al. 2019).

The current study took place in Finland where children first attend to one-year pre-primary education (at age 6) and then continue their studies in compulsory nine-year basic education which consists of primary school (Grades 1–6) and lower secondary school (Grades 7–9). Primary school grades and lower secondary school grades are typically housed separately but also other types exist (e.g., approximately every fifth school is a joint school comprising Grades 1 to 9). After compulsory basic education students can choose between different options, the most common ones being general upper secondary education (i.e., academic track), vocational education and training (i.e., vocational track), and combinations of vocational and academic track studies. Typically, approximately 50% of Finnish students select general upper secondary education and approximately 40% choose vocational education and training, while the other options are less typical (Official Statistics of Finland 2018). Upper secondary education studies typically take three to four years to complete and after graduation students are eligible to apply for higher education.

## Methods

### Participants and Procedure

The data used in the present study were drawn from the First Steps follow-up study (Lerkkanen et al. 2006–2016) and its extension, that is, the School Path: From First Steps to Secondary and Higher Education study (Vasalampi and Aunola 2016–2020). The First Steps follow-up study started in 2006. Approximately 2000 children born in 2000 were followed up 10 times from kindergarten to the end of lower secondary school (Grade 9) in four Finnish municipalities. In the School Path: From First Steps to Secondary and Higher Education study, the participants were followed up twice during upper secondary education. In the present study, data from four time points that extended across the two phases of the follow-up were used. The time points focused on the following critical transition phases: data collection for Time 1 took place at the end of primary school (Grade 6, 12 years of age, April 2013), Time 2 in the first year of lower secondary school (Grade 7, 13 years of age, April 2014), Time 3 in the final year of lower secondary school (Grade 9, 15 year of age, April 2016), and Time 4 in the first year of upper secondary education (i.e., Grade 1 in upper secondary education, 16 years of age, January through March 2017). Students participated during normal school lessons. Trained researcher assistants administered the questionnaires in the classrooms at T1–T3 and trained research assistants or teachers at T4. At Time 1 (Grade 6), group-administered paper-and-pencil reading and arithmetic tests for sixth graders were conducted in the classrooms in addition to questionnaires. Written informed consent was obtained from participants’ parents or guardians for data collection in primary and lower secondary school (T1–T3), and the upper secondary education participants provided informed written consent to confirm their voluntary participation in the study (T4).

A total of 2082 students participated in the follow-up study at least once from T1 to T4. As the main focus was on school burnout in upper secondary education, only students who participated in Time 4 and answered the questionnaire regarding school burnout in Time 4 were included in the analyses. The final data comprise 1544 students, which is 74.2% of all available data. At T1, the number of participants was 1298 (50.0% boys, mean age 12.74, *SD* = 0.31), at T2 1274 (49.0% boys, mean age 13.75, *SD* = 0.31), at T3 1292 (49.6% boys, mean age 15.73, *SD* = 0.32), and at T4 1544 (48.7% boys, mean age 16.66, *SD* = 0.36). At T4, 986 (63.9%) participants were enrolled in the academic track (general upper secondary schools), 470 (30.4%) in the vocational track (vocational institutions), 78 (5.1%) in double-degree programs (i.e., a combination of the academic and vocational tracks), and 10 (0.6%) in post-comprehensive programs that do not lead to an upper secondary education certificate (e.g., extra Grade 10 or folk high school). Students who were absent during data collection at T4 were later contacted personally. Although 38 later contacted students (2.5% of the sample) were already attending the second year of upper secondary education when they answered the questionnaire, they were still included in the sample.

Attrition analyses were conducted to make comparisons between participants who were excluded from the analyses because of missing data at T4 (*n* = 538) and those whose data were included in the analyses (*n* = 1544). These analyses indicated that students who were excluded showed statistically significantly lower levels of arithmetic skills, reading skills, and self-esteem in Grade 6 and higher levels of cynicism in Grades 6, 7, and 9 than those who were included.

### Measures

#### School burnout

Two symptoms of school burnout were assessed in the present study, that is, exhaustion and cynicism using six items drawn from the School Burnout Inventory (SBI, Salmela-Aro et al. 2009a). Originally the SBI (Salmela-Aro and Näätänen 2005) was developed from the Bergen Burnout Indicator 15 (Näätänen et al. 2003) by changing the work context to the school context. The SBI has shown good reliability and validity (Salmela-Aro et al. 2009a). Three of the items measured exhaustion (“I often sleep badly because of matters related to my schoolwork”; “I brood over matters related to my schoolwork a lot during my free time”; “The pressure of my schoolwork disturbs my life outside school^1^”), and three of the items measured cynicism (“I feel a lack of motivation in my schoolwork^2^”; “I feel that I am losing interest in my schoolwork”; “I’m continually wondering whether my schoolwork has any meaning”). Adolescents were asked to rate each item on a 5-point Likert scale (1 = completely disagree; 5 = completely agree). A composite score was calculated separately for exhaustion and cynicism for each measurement point as the mean of the items measuring the constructs. The Cronbach’s alpha reliability coefficients for exhaustion were 0.816, 0.831, 0.857, and 0.864 for T1, T2, T3 and T4, respectively. Cronbach’s alpha reliability coefficients for cynicism were 0.832, 0.850, 0.846, and 0.852 for T1, T2, T3, and T4, respectively.

#### Internalizing symptoms

Internalizing symptoms at T1 were assessed with the Finnish version of the Strengths and Difficulties Questionnaire (SDQ; Goodman 1997; Koskelainen et al. 2000). The SDQ consists of five subscales of students’ self-ratings: conduct problems, hyperactivity/inattention, emotional symptoms, peer problems, and prosocial behavior. The scale for emotional symptoms was used as a measure of internalizing symptoms. Five items measure internalizing symptoms during the previous six months in Grade 6 (e.g., “I worry a lot”). A 3-point Likert scale rates the items (1 = not true, 2 = somewhat true, 3 = certainly true). A composite score was calculated as the mean of the items. The Cronbach’s alpha reliability coefficient for internalizing symptoms was 0.682.

#### Self-esteem

Self-esteem at T1 was assessed with a shortened version of the Rosenberg Self-Esteem Scale (Rosenberg 1965). Three self-report items measure self-esteem (e.g., “On the whole, I am satisfied with myself”). A 5-point Likert scale rates the items (1 = totally disagree; 5 = totally agree). A composite score was calculated as a mean of the items. The Cronbach’s alpha reliability coefficient for self-esteem was 0.872.

#### Reading skills

Reading skills were assessed at T1 using three group-administered tests: a word-reading fluency task, a word-chain task, and a sentence-level reading fluency task. The word-reading fluency task is a subtest of the nationally normed reading test battery (ALLU; Lindeman 2000). The task comprises 80 items each of which includes a picture with four phonologically similar words attached to it. Students are asked to silently read the words and then to draw a line to semantically match the correct word with the picture. The task has a two-minute time limit, and the score is the number of correct answers (maximum of 80).

The word-chain task (Nevala and Lyytinen 2000) comprised 10 rows of word chains which are formed from 4 to 6 words written together. Students are asked to silently read the word chains and while reading them, indicate the word boundaries by drawing a dividing line in between the words. The task has a one-minute time limit, and the score is the number of correct responses (maximum of 40).

Sentence-level reading fluency was assessed using the Finnish version of the Salzburger Lese-Screening Test (SLS; Mayringer and Wimmer 2003). The task comprises 69 sentences which are either true or false (e.g., “Blueberries are yellow”). Students are asked to read the sentences silently and verify the truthfulness of the sentences. The task has a two-minute time limit, and the score is the number of correct answers (maximum of 69).

A composite score for reading skills was calculated as the mean of the three standardized test scores. The Cronbach’s alpha reliability coefficient for reading skills was 0.779.

#### Arithmetic skills

Arithmetic skills were assessed at T1 with the group-administered Basic Arithmetic test (Aunola and Räsänen 2007) which consists of 28 addition, subtraction, multiplication, and division tasks (e.g., 40: 8 – 3 = __?; __ – 18 = 45 – 12?). The task increases in difficulty and requires accuracy and speed (i.e., automatization of basic calculation routines). The task has a three-minute time limit, and the score is the number of correct answers (maximum of 28). The arithmetic skills variable was standardized and forced to the range of −3 to 3 to ensure that possible outliers would not impact the results.

### Data Analyses

Statistical analyses were performed using latent growth curve modeling (LGM). Linear LGM estimates two growth components, that is, the level (defined as the initial level in the present study) component and the slope (i.e., linear trend) component. By estimating the means and variances of these growth components, LGM examines not only intra-individual changes (comparable to repeated-measures ANOVA) of the construct of interest but also inter-individual differences in the initial level and in the developmental trend. In other words, LGM analyses consider the possibility that the initial levels and developmental trends differ between individuals. When using LGM, it is therefore possible to examine various predictors and outcomes of the individual variation in growth components as well.

The LGM analyses were conducted in three steps, separately for exhaustion and cynicism. First, LGM across three measurement points, that is, Grade 6 (T1), Grade 7 (T2), and Grade 9 (T3), was carried out. For the level component, the loadings of the observed burnout variables across T1 to T3 were fixed to 1, whereas the slope component loadings were fixed to 0, 1, and 3, respectively, representing the linear slope across the three measurement points. The residual variances were freely estimated and the level and slope components were allowed to correlate. Second, to investigate whether the initial level of school burnout symptoms in Grade 6 (T1), and the developmental trend (i.e., linear slope T1–T3) predict students’ school burnout symptoms in the first year of upper secondary education (T4), the latent variable of exhaustion/cynicism was added to the previous model as an outcome variable, and the level and slope components predicted the level of this latent variable. The residual variances of the observed exhaustion and cynicism variables (T4) were estimated freely. At this stage the path from the level to the slope was estimated to control for the impact of the level on the slope. Third, the hypothesized antecedents of school burnout symptoms (i.e., reading skills, arithmetic skills, internalizing symptoms, self-esteem, and gender) measured at T1 were added to the previous model as covariates and direct and indirect paths (i.e., via level and slope) from the antecedents to school burnout symptoms in upper secondary education were modeled. Each antecedent was added to the model separately. Then, variables demonstrating statistically significant paths were included in the same model to test their unique impacts over and above other covariates. After this, non-statistically significant paths were excluded from the final model.

The analyses were conducted using the Mplus statistical package (Version 8.4, Muthén and Muthén 1998–2017). The models were estimated using the MLR estimator with a missing data method, which uses all available observations in the data to estimate the parameters of the model. The goodness-of-fit of the LGM models was evaluated via five indicators: chi-square test, comparative fit index (CFI), Tucker–Lewis index (TLI), root mean square error of approximation (RMSEA), and standardized root mean square residual (SRMR).

## Results

### Exhaustion

The means (*M*), standard deviations (*SD*), and correlations between study variables are shown in Table 1.

**Table 1:** Means (*M*) and standard deviations (*SD*) of the study variables and correlation matrix

	*M*	*SD*	1	2	3	4	5	6	7	8	9	10	11	12	13	14	15	16	17
1. Exhaustion T1	2.00	0.89	1																
2. Exhaustion T2	2.11	0.93	0.50***	1															
3. Exhaustion T3	2.40	1.04	0.39***	0.44***	1														
4. Exhaustion T4/item 1	2.17	1.11	0.25***	0.28***	0.47***	1													
5. Exhaustion T4/item 2	2.67	1.28	0.30***	0.32***	0.50***	0.64***	1												
6. Exhaustion T4/item 3	2.30	1.17	0.26***	0.31***	0.49***	0.64***	0.77***	1											
7. Cynicism T1	2.23	0.94	0.38***	0.19***	0.08**	0.03	−0.02	0.01	1										
8. Cynicism T2	2.22	0.95	0.23***	0.37***	0.11***	0.01	−0.04	0.03	0.58***	1									
9. Cynicism T3	2.27	0.98	0.16***	0.21***	0.33***	0.12***	0.02	0.10***	0.42***	0.51***	1								
10. Cynicism T4/item 1	1.89	0.93	0.10***	0.13***	0.19***	0.28***	0.19***	0.30***	0.26***	0.28***	0.45***	1							
11. Cynicism T4/item 2	2.06	1.08	0.15***	0.16***	0.31***	0.44***	0.37***	0.47***	0.19***	0.18***	0.36***	0.69***	1						
12. Cynicism T4/item 3	1.80	1.02	0.14***	0.17***	0.28***	0.36***	0.33***	0.40***	0.17***	0.18***	0.35***	0.60***	0.69***	1					
13. Reading skills	0.00	0.83	0.00	−0.03	0.07*	0.03	0.10***	0.05	−0.02	−0.05	−0.01	0.01	0.09**	0.06*	1				
14. Arithmetic skills	0.00	1.00	−0.12***	−0.09**	−0.09**	−0.07*	0.00	−0.01	−0.10***	−0.10***	−0.12***	−0.07**	−0.04	−0.06*	0.44***	1			
15. Internalizing symptoms	1.51	0.42	0.47***	0.33***	0.29***	0.22***	0.27***	0.22***	0.25***	0.19***	0.15***	0.10***	0.16***	0.16***	0.06*	−0.10**	1		
16. Self-esteem	3.70	0.79	−0.36***	−0.25***	−0.17***	−0.16***	−0.15***	−0.12***	−0.38***	−0.31***	−0.19***	−0.14***	−0.13***	−0.13***	0.01	0.20***	−0.41***	1	
17. Gender			−0.10***	−0.11***	−0.30***	−0.21***	−0.35***	−0.25***	0.13***	0.12***	0.08**	0.02	−0.08**	−0.05*	−0.24***	0.09**	−0.25***	0.21***	1

*p < 0.05; **p < 0.01; ***p < 0.001

LGM across three measurement points T1, T2, and T3, that is, Grade 6, Grade 7, and Grade 9, was estimated for exhaustion. Based on goodness-of-fit indicators, the estimated linear model fit the data well (*χ*^2^ (1, 1357) = 0.479, *p* = 0.489, CFI = 1, TLI = 1, RMSEA = 0.000, SRMR = 0.005). The results are presented in Table 2. The mean of the slope of exhaustion was positive and statistically significant indicating that, on average, exhaustion increased among students from Grade 6 to Grade 9. The results showed further that the variances of both growth components were statistically significant suggesting that there were individual differences in the level and in the slope of exhaustion.

**Table 2:** Unstandardized parameter estimates of the tested unconditional latent growth curve models (*n* = 1357)

	Means		Variances			Residual variances		
	Level	Slope	Level	Slope	Covariance (level, slope)	Time 1	Time 2	Time 3
	Estimate (S.E.)	Estimate (S.E.)	Estimate (S.E.)	Estimate (S.E.)	Estimate (S.E.)	Estimate (S.E.)	Estimate (S.E.)	Estimate (S.E.)
Model 1: exhaustion	1.989*** (0.023)	0.137*** (0.010)	0.440*** (0.037)	0.033** (0.013)	-0.027* (0.013)	0.348*** (0.036)	0.454*** (0.028)	0.507*** (0.081)
Model 2: cynicism	2.224*** (0.025)	0.014 (0.010)	0.570*** (0.040)	0.051*** (0.012)	-0.062*** (0.014)	0.306*** (0.036)	0.401*** (0.028)	0.301*** (0.076)

*p < 0.05; **p < 0.01; ***p < 0.001

Next, to examine whether the initial level and slope of exhaustion predict subsequent exhaustion in upper secondary education (H1 and H2), the level of exhaustion in the first year of upper secondary education (T4) was added to the previous model as a latent outcome variable and paths from the level and slope components of exhaustion to this outcome variable were estimated. The residual variances of the observed exhaustion variables were first freely estimated, but due to non-identification of the model, the residual variances of T2 and T3 were set to be equal. The model fit the data well (*χ*^2^ (9, 1544) = 18.307, *p* = 0.032, CFI = 0.997, TLI = 0.994, RMSEA = 0.026, SRMR = 0.017). The results showed that the level and slope of exhaustion statistically significantly predicted exhaustion in upper secondary education (T4): the higher the level of exhaustion in Grade 6, the higher the level of exhaustion in upper secondary education (standardized estimate = 0.559, *SE* = 0.051, *p* < 0.001). Additionally, the more exhaustion increased during the period between T1 and T3, the higher the level of exhaustion in upper secondary education (standardized estimate = 0.678, *SE* = 0.053, *p* < 0.001).

**Fig. 1:**
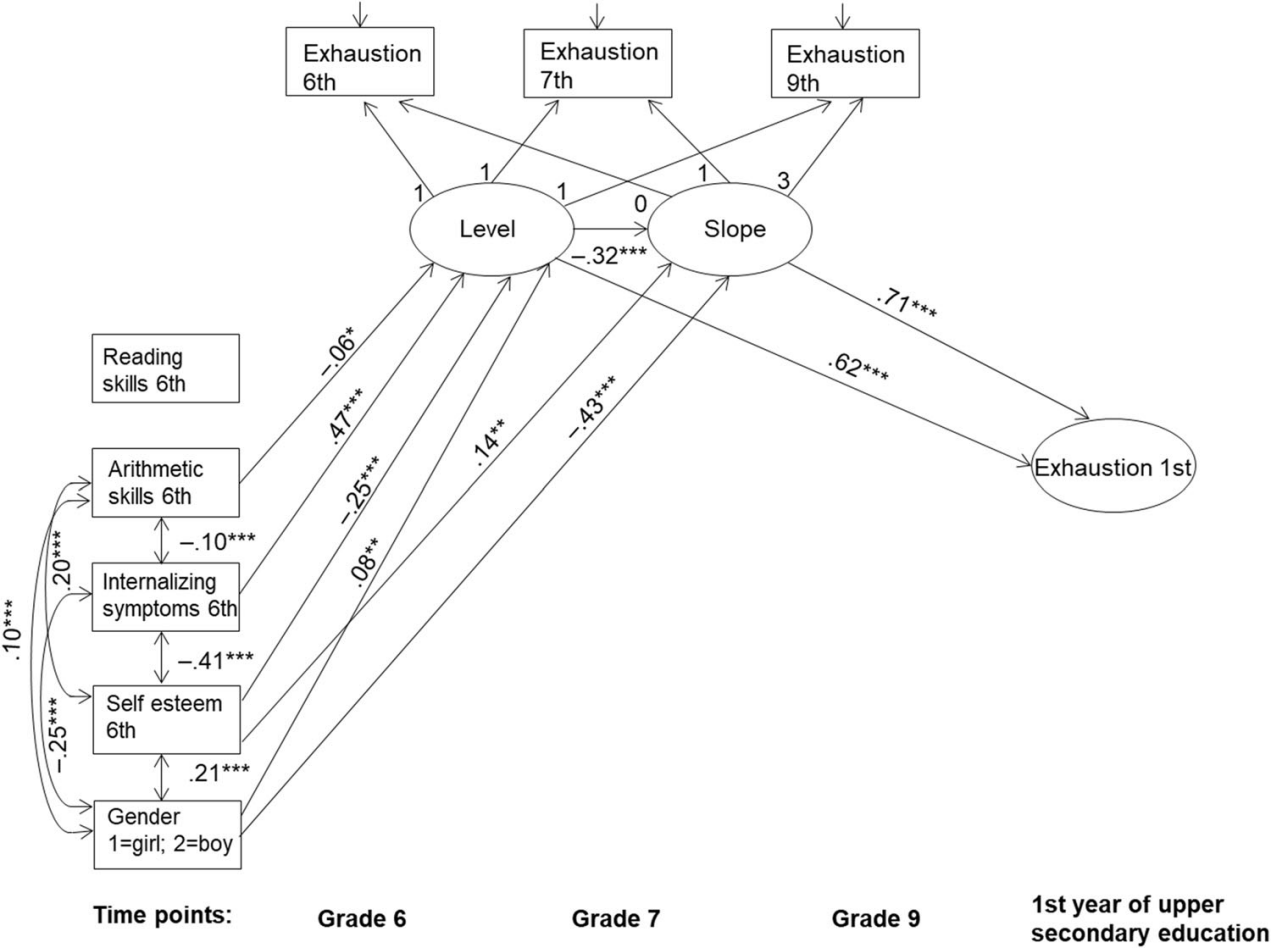
Final LGM model of exhaustion with covariates. Only statistically significant paths are presented. Estimates are shown as standardized estimates. *p < 0.05; **p < 0.01; ***p < 0.001

Finally, the potential antecedents, that is, reading skills, arithmetic skills, internalizing symptoms, self-esteem, and gender were added to the previous model as covariates. The aim was to examine whether these antecedents predict individual differences in the level of exhaustion in upper secondary education either directly or indirectly via individual differences in the level and slope components of exhaustion (H3 and H4). The final model including only the statistically significant paths fit the data well (*χ*^2^ (27, 1544) = 100.436, *p* < 0.001, CFI = 0.979, TLI = 0.969, RMSEA = 0.042, SRMR = 0.024) and is presented in Fig. 1. The results (Fig. 1) showed that none of the antecedents directly predicted the level of exhaustion in upper secondary education, whereas indirect effects were found.

The results (Table 3) showed first that students’ level of arithmetic skills had a statistically significant indirect negative effect on exhaustion in upper secondary education via the level of exhaustion in Grade 6: the lower the levels of arithmetic skills students had in Grade 6, the higher the levels of exhaustion they reported in Grade 6 and consequently, the higher the level of exhaustion they reported in upper secondary education. Second, the results (Table 3) showed that students’ internalizing symptoms in Grade 6 had a statistically significant indirect effect on exhaustion in upper secondary education via the initial level of exhaustion, the total indirect effect being positive and statistically significant: the higher the levels of internalizing symptoms students showed in Grade 6, the higher the levels of exhaustion the students reported in Grade 6 and consequently, the higher the levels of exhaustion they showed in upper secondary education.

**Table 3:** Specific indirect effects, total indirect effects, and total effects of the covariates to upper secondary education exhaustion and cynicism

	Exhaustion in upper secondary education (T4)					Cynicism in upper secondary education (T4)				
	Indirect effect					Indirect effect				
	Via level	Via slope	Via level and slope	Total indirect	Total	Via level	Via slope	Via level and slope	Total indirect	Total
Covariate (T1)	Estimate (S.E.)	Estimate (S.E.)	Estimate (S.E.)	Estimate (S.E.)	Estimate (S.E.)	Estimate (S.E.)	Estimate (S.E.)	Estimate (S.E.)	Estimate (S.E.)	Estimate (S.E.)
Reading skills	–	–	–	–	–	–	–	–	–	0.062* (0.029)
Arithmetic skills	−0.036* (0.018)	–	0.013 (0.007)	−0.023* (0.012)	−0.023* (0.012)	–	−0.039* (0.016)	–	−0.039* (0.016)	−0.039* (0.016)
Internalizing symptoms	0.295*** (0.032)	–	−0.106*** (0.027)	0.189*** (0.022)	0.189*** (0.022)	0.091*** (0.018)	–	−0.035*** (0.009)	0.055*** (0.013)	0.055*** (0.013)
Self-esteem	−0.153*** (0.025)	0.099** (0.033)	0.055*** (0.014)	0.001 (0.029)	0.001 (0.029)	−0.206*** (0.023)	–	0.080*** (0.016)	−0.126*** (0.018)	−0.126*** (0.018)
Gender	0.051** (0.018)	−0.302*** (0.027)	−0.018* (0.007)	−0.269*** (0.027)	−0.269*** (0.027)	0.142*** (0.018)	–	−0.055*** (0.011)	0.087*** (0.014)	−0.006 (0.028)

Estimates are shown as standardized estimates (standard errors in parentheses)

*p < 0.05; **p < 0.01; ***p < 0.001

Third, gender had a statistically significant indirect effect on exhaustion in upper secondary education via the slope of exhaustion (Table 3): girls showed a higher increase in exhaustion across lower secondary school than boys, and consequently, girls reported higher levels of exhaustion than boys in upper secondary education. In the final model, the path from gender on the initial level of exhaustion in Grade 6 was statistically significant and positive (Fig. 1) as well as the related indirect effect (Table 3). However, when gender was included in the model as the only predictor variable, the effect was negative. Thus, the positive effect of gender on the level of exhaustion in Grade 6 in the final model might reflect a suppressor effect.

Finally, the results showed (Fig. 1) that students’ level of self-esteem in Grade 6 had a negative effect on the concurrent level of exhaustion in Grade 6 and a positive effect on the slope of exhaustion, but the total indirect effect of students’ self-esteem on their exhaustion in upper secondary education was not statistically significant (Table 3). In other words, the lower the levels of self-esteem students showed in Grade 6, the higher the levels of exhaustion they reported in Grade 6 but the less exhaustion increased across lower secondary school. However, due to the compensatory effects, the total indirect effect on subsequent exhaustion in upper secondary education was not statistically significant.

### Cynicism

LGM across three measurement points T1, T2, and T3, that is, Grade 6, Grade 7, and Grade 9, was carried out for cynicism. Based on the goodness-of-fit indicators, the estimated linear model fit the data well (*χ*^2^ (1, 1357) = 1.342, *p* = 0.247, CFI = 0.999, TLI = 0.998, RMSEA = 0.016, SRMR = 0.007). The results are presented in Table 2. The mean of the slope of cynicism was not statistically significant indicating that, on average, there was no change in cynicism among the students from Grade 6 to Grade 9. The results showed further that the variances of both growth components were statistically significant (Table 2): there were individual differences in the level and in the slope of cynicism.

Next, to examine whether the initial level and slope of cynicism predict subsequent cynicism in upper secondary education (H1 and H2), the level of cynicism in upper secondary education (T4) was added to the previous model as a latent outcome variable and paths from the level and slope components of cynicism to this outcome variable were estimated. The model fit the data well (*χ*^2^ (8, 1544) = 60.124, *p* < 0.001, CFI = 0.977, TLI = 0.957, RMSEA = 0.065, SRMR = 0.031). The results showed that the level and slope of cynicism statistically significantly predicted the subsequent cynicism in upper secondary education (T4): the higher the level of students’ cynicism was in Grade 6, the higher the level of students’ cynicism in upper secondary education (standardized estimate = 0.448, *SE* = 0.042, *p* < 0.001). Additionally, the more cynicism increased during the study period, the higher the level of cynicism in upper secondary education (standardized estimate = 0.474, *SE* = 0.056, *p* < 0.001).

**Fig. 2:**
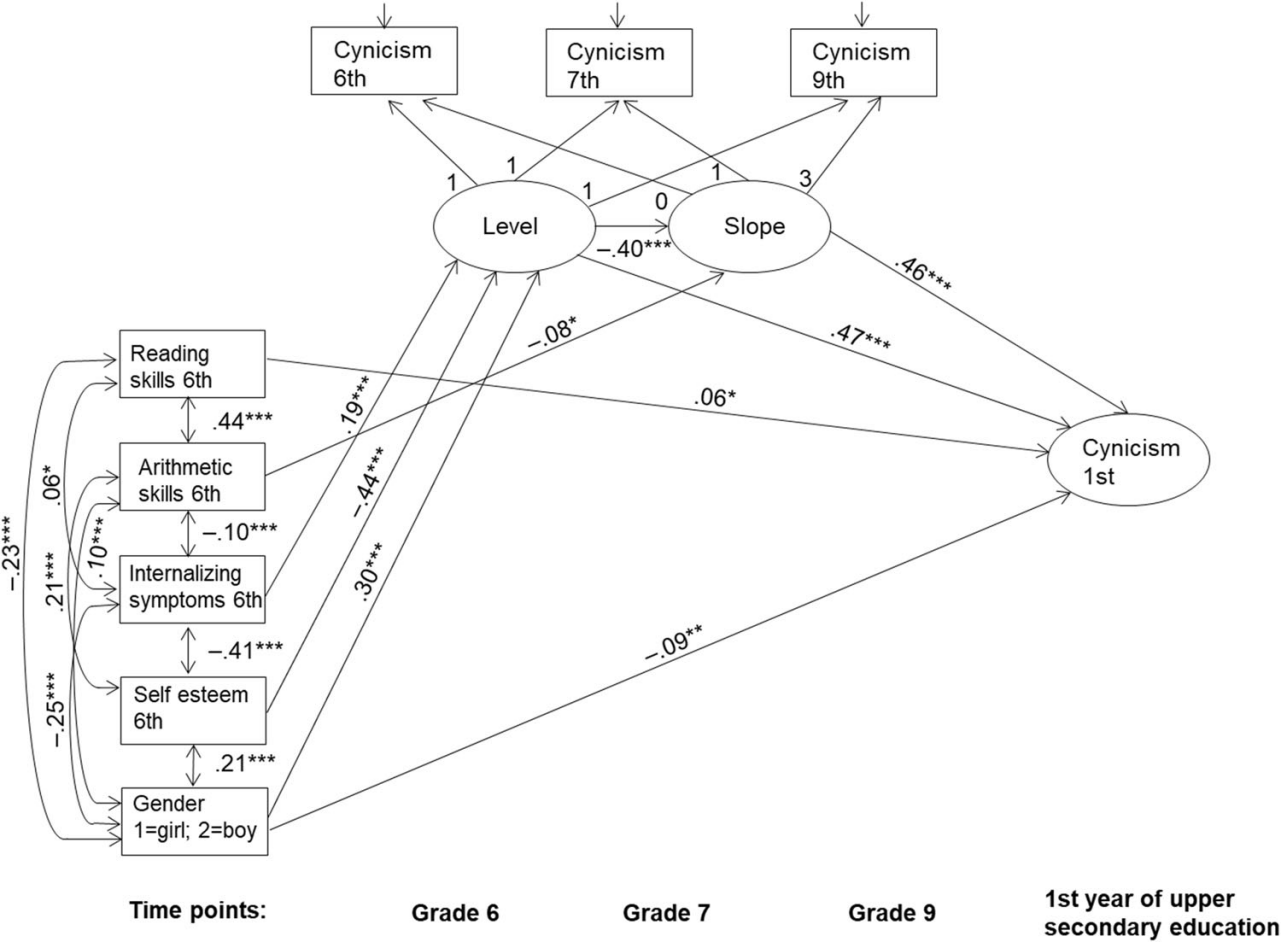
Final LGM model of cynicism with covariates. Only statistically significant paths are presented. Estimates are shown as standardized estimates. *p < 0.05; **p < 0.01; ***p < 0.001

Finally, the potential antecedents, that is, reading skills, arithmetic skills, internalizing symptoms, self-esteem, and gender, were added to the LGM model as covariates. The aim was to examine whether the antecedents predict individual differences in the level of cynicism in upper secondary education either directly or indirectly via the level and slope components of cynicism (H3 and H4). The final model including only statistically significant paths fit the data well (*χ*^2^ (32, 1544) = 114.661, *p* < 0.001, CFI = 0.973, TLI = 0.962, RMSEA = 0.041, SRMR = 0.027) and is presented in Fig. 2.

The results showed (Fig. 2), first, that the level of reading skills in Grade 6 directly predicted the level of cynicism in the first year of upper secondary education: the higher the levels of reading skills students demonstrated in Grade 6, the higher the level of cynicism they reported in upper secondary education. Second, the results (Table 3) showed an indirect negative effect from Grade 6 arithmetic skills to students’ cynicism in upper secondary education: the lower the students’ level of arithmetic skills in Grade 6, the more their cynicism increased across lower secondary school and consequently, the higher the level of cynicism they reported in upper secondary education. Third, the results (Table 3) showed that students’ level of internalizing symptoms in Grade 6 had an indirect effect on their cynicism in upper secondary education via the level of cynicism in Grade 6, the total indirect effect being positive: the higher the levels of internalizing symptoms students showed in Grade 6, the higher the levels of cynicism they reported in Grade 6 and consequently, the higher the levels of cynicism they showed in upper secondary education.

Fourth, the results (Table 3) showed that there was an indirect effect of students’ self-esteem on cynicism in upper secondary education via the level of cynicism at Grade 6, the total indirect effect being statistically significant and negative: the lower the level of self-esteem students showed in Grade 6, the higher the levels of cynicism they reported in Grade 6 and consequently, the higher the level of cynicism they reported in upper secondary education. Fifth, the results (Fig. 2) showed that although gender had a negative direct effect on cynicism suggesting that girls reported higher levels of cynicism in upper secondary education than boys, due to the opposite indirect effect of gender on cynicism via the initial level of cynicism (Table 3), the total effect of gender on upper secondary education cynicism was not statistically significant. In other words, taking into account all the effects, no gender differences were evident in cynicism in upper secondary education.

## Discussion

Previous literature has documented that school burnout is a prevalent and alarming problem globally among students attending upper secondary education (Walburg 2014). Although the need to tackle school burnout is widely acknowledged, little research is available which would shed on the developmental trajectories and antecedents that underlie school burnout symptoms. Therefore, the present study examined the extent to which school burnout symptoms in upper secondary education may have roots in primary and lower secondary school and whether various early antecedents of school burnout could be identified using a comprehensive longitudinal data set spanning four time points and two transitions.

The results for exhaustion and cynicism showed, as hypothesized (H1), that the higher level of symptoms students reported at the final year of primary school, the higher their symptoms in the first year of upper secondary education. In addition to the initial level of school burnout symptoms in primary school, the increase in exhaustion and cynicism across the period from the end of primary school through lower secondary school was associated with higher levels of symptoms in upper secondary education (H2). Previous studies have detected signs of school burnout among primary school students (Salmela-Aro et al. 2016; Yang and Chen 2016) and have found that symptoms increase over the course of school years (Engels et al. 2019; Lee and Lee 2018). The present longitudinal results spanning a five-year period showed continuity in students’ school burnout symptoms and suggest that early signs of symptoms and adverse developmental trajectories should be taken seriously as they can indicate considerable risk for subsequent school burnout symptoms in upper secondary education. The end of primary school as well as lower secondary school years may be critical time windows for school-wide and individual implementation of school burnout intervention actions.

The present study showed that various early predictors of upper secondary education student burnout can be identified in primary school. Especially the internalizing symptoms in the end of primary school may constitute risk factors for experiencing school burnout symptoms in upper secondary education. In line with the hypothesis (H3), the present study showed that the higher levels of internalizing symptoms students showed in Grade 6, the higher the levels of exhaustion and cynicism they reported in Grade 6 and consequently, the higher the levels of these school burnout symptoms they showed in upper secondary education. The finding concurs with previous studies that showed students’ school burnout symptoms coincide with problems of psychological well-being (Parviainen et al. 2020; Virtanen et al. 2019). The present study extended these previous findings by showing that internalizing symptoms experienced at age of 12–13 years were associated with school burnout symptoms four years later in a different context, in upper secondary education. The findings suggest that students who experience higher levels of internalizing symptoms may be more likely to react to school-related overload and experience school burnout symptoms.

The role of self-esteem, another indicator of psychological well-being, was less prominent than that of internalizing symptoms in predicting school burnout symptoms. Previous studies have linked lower self-esteem with higher levels of school burnout symptoms (Kiuru et al. 2008; Luo et al. 2016). In the present study, as hypothesized (H3), the lower the level of self-esteem students showed in Grade 6, the higher the levels of cynicism they also reported in Grade 6 and consequently, the higher the level of cynicism they reported in upper secondary education. In contrast, although a higher level of exhaustion in Grade 6 was linked to lower self-esteem in Grade 6, the level of self-esteem did not statistically significantly predict exhaustion four years later in upper secondary education. The results suggest that self-esteem is associated only with subsequent cynicism but not to the same extent with exhaustion. Thus, it is possible that the level of self-esteem in primary school is neither a risk nor a protective factor for exhaustion in upper secondary education. With respect to cynicism, it is possible that students who have negative perceptions of themselves may, for example, question their ability to succeed in school, and have negative thoughts about the value of school which could lead to the development of a cynical attitude toward studying. However, more studies are needed to examine the mechanisms behind this relation.

Although researchers have linked higher school burnout symptoms to lower academic achievement (Madigan and Curran 2020), scholars (Korhonen et al. 2014; Widlund et al. 2018) have shown that the link between academic skills and school burnout is not straightforward, which was also detected in the present study. The results showed, as hypothesized (H3), that lower levels of arithmetic skills in Grade 6 indirectly predicted higher levels of exhaustion and cynicism in upper secondary education. The lower the levels of arithmetic skills students had at Grade 6, the higher the levels of exhaustion they reported in Grade 6 and consequently, the higher the level of exhaustion they reported in upper secondary education. Additionally, the lower the students’ level of arithmetic skills in Grade 6, the more their cynicism increased across lower secondary school and consequently, the higher the level of cynicism they reported in upper secondary education. Students with poorer arithmetic skills may be at risk for school burnout symptoms because struggles in this key subject with high relevance for further educational choices may increase school-related exhaustion and an indifferent, cynical attitude toward school. In contrast to the hypothesis (H3), better reading skills in Grade 6 predicted directly higher levels of cynicism in upper secondary education. This finding indicates that students who have better reading skills in Grade 6 experience higher levels of cynicism in upper secondary education. This finding concurs with previous studies which suggest that students who perform relatively well in school can be at risk for school burnout (Korhonen et al. 2014). Some good readers may experience the demands of upper secondary education as overtaxing, and their motivation may wane. Overall, these findings suggest that students with various skill levels can be likely to experience school burnout.

In addition, gender differences in school burnout symptoms were found. Previous studies have shown that girls are more likely to experience exhaustion than boys (Herrmann et al. 2019; Salmela-Aro et al. 2008a), but gender differences are less prominent for cynicism (Cadime et al. 2016; Herrmann et al. 2019). In the present study, gender predicted school burnout symptoms in upper secondary education only with respect to exhaustion but not cynicism. As hypothesized (H4), girls showed higher levels of exhaustion in upper secondary education. Scholars have found that girls experience high school-related pressure and demands (Wiklund et al. 2012), and it can be speculated that higher levels of exhaustion among girls may be linked, for example, to the experience of gendered demands for hard-working and conscientious study orientation exposing girls more often to exhaustion. However, further research is needed to understand the development of exhaustion among girls and boys.

### Practical Implications

The present results call for preventive actions before upper secondary education and suggest that attention might be good to direct to earlier education phases. For students for whom antecedents and thus, risk factors of school burnout accumulate during the early years of education, it may be more difficult and more critical to find effective ways to break the cycle of adverse development. Tackling development of school burnout early on may prevent burnout symptoms not only in upper secondary education but also in tertiary education and during the transition to work-life. Implementation of tailored and timely interventions can be promoted by better recognition of signs of increased susceptibility to school burnout symptoms. The present study highlights that attention should be paid especially to students who manifest higher levels of internalizing symptoms, lower self-esteem, and poorer arithmetic skills at the end phase of primary school. These students might need more support for study skills and managing school-related demands and pressure. However, also students who show good reading skills and self-esteem may need support in particular at the beginning of upper secondary education when expectations for autonomous study work and independent decision making increase. Because the anteceding factors of school burnout studied in the present study were partly differently associated with the development of exhaustion and cynicism, their unique characteristics and potentially different meanings for girls and boys should be considered in designing school burnout prevention strategies. Scholars have found that school-based interventions aiming to reduce psychological stress are effective among selected students (for review, see van Loon et al. 2020). However, general support for preventing school burnout should be made available to all students in line with evidence that, for example, supportive educational practices (Meylan et al. 2020) and motivating teachers (Salmela-Aro et al. 2008b) are linked to lower school burnout symptoms among students.

### Limitations and Suggestions for Future Research

The present study increased understanding of the roots of upper secondary education students’ school burnout, but a few limitations leave room for future research. First, the present study included only three measurement points of school burnout symptoms at comprehensive school and thus linear model was used to describe the development of school burnout symptoms. It is possible, however, that with more measurement points other forms of change could be evident (e.g., quadratic growth). Therefore, future studies with more measurement points are needed to model the growth of school burnout symptoms across comprehensive school. Second, the third school burnout dimension, inadequacy, was not examined. In further studies, the development of inadequacy, as well as antecedents of it, should be investigated. Third, the reliability of internalizing symptoms measure was relatively low. Consequently, replications of the results using alternative instrument to assess internalizing symptoms are needed. Fourth, some paths from the antecedents to upper secondary education school burnout symptoms were only barely significant and firm conclusions could not be drawn. In particular, researchers should examine the role of academic skills in school burnout in further studies. In the present study, academic skills were examined solely as a continuum of skills drawn from standardized testing, and it would be relevant to investigate whether identified learning difficulties are linked to school burnout symptoms and their trajectories. Fifth, only a few potential individual-level antecedents of school burnout were examined, and in future studies, researchers could include a broader set of individual- and environmental-level risk and protective factors in the design. It would be relevant to examine what makes some students resilient to the negative effects of school burnout and why school burnout symptoms are more detrimental to some students. Finally, the present study showed that there was individual variation in the trajectories of school burnout symptoms, opening up an evident need for person-centered studies on the development of school burnout symptoms in the future. Further research is needed to examine whether a subgroup of students who show high levels of symptoms continuously from primary school to upper secondary education can be identified, and which factors might cause the high levels of symptoms early on. In order to identify at-risk students, clinical thresholds for school burnout should be developed and tested in future studies.

## Conclusion

There is consensus that school burnout among students attending upper secondary education is a concerning problem that should be tackled as early as possible. However, thus far the understanding of the background of the school burnout symptoms has been limited because of lack of longitudinal studies. The findings of the present longitudinal study demonstrated that school burnout symptoms in upper secondary education may have roots in earlier education phases as the level of school burnout symptoms at the end of primary school and the increase in these symptoms across the transition from primary school through lower secondary school predicted the level of school burnout symptoms in upper secondary education. Moreover, psychological well-being, academic skills, and gender were found to contribute to the prediction of school burnout symptoms. Overall, this study strengthens the idea that attention should be directed already to primary and lower secondary school years and potential warning signs of school burnout should be recognized before upper secondary education.

## Footnotes

1 The item was slightly modified from the original in order to make the item suitable for the context of the present study. The original item was “The pressure of my schoolwork causes me problems in my close relationships with others”

2 The item was slightly modified from the original in order to make the item suitable for the context of the present study. The original item was: “I feel a lack of motivation in my schoolwork and often think of giving up.”
